# Effect of Predeparture Testing on Postarrival SARS-CoV-2–Positive Test Results Among International Travelers — CDC Traveler-Based Genomic Surveillance Program, Four U.S. Airports, March–September 2022

**DOI:** 10.15585/mmwr.mm7208a2

**Published:** 2023-02-24

**Authors:** Stephen M. Bart, Teresa C. Smith, Sarah Anne J. Guagliardo, Allison Taylor Walker, Benjamin H. Rome, Siyao Lisa Li, Thomas W. S. Aichele, Rob Stein, Ezra T. Ernst, Robert C. Morfino, Martin S. Cetron, Cindy R. Friedman

**Affiliations:** ^1^Division of Global Migration and Quarantine, National Center for Emerging and Zoonotic Infectious Diseases, CDC; ^2^Ginkgo Bioworks, Inc., Boston, Massachusetts; ^3^XpresCheck, XWELL, New York, New York.

Beginning December 6, 2021, all international air passengers boarding flights to the United States were required to show either a negative result from a SARS-CoV-2 viral test taken ≤1 day before departure or proof of recovery from COVID-19 within the preceding 90 days (*1*). As of June 12, 2022, predeparture testing was no longer mandatory but remained recommended by CDC (*2*,*3*). Various modeling studies have estimated that predeparture testing the day before or the day of air travel reduces transmission or importation of SARS-CoV-2 by 31%–76% (*4*–*7*). Postarrival SARS-CoV-2 pooled testing data from CDC’s Traveler-based Genomic Surveillance program were used to compare SARS-CoV-2 test results among volunteer travelers arriving at four U.S. airports during two 12-week periods: March 20–June 11, 2022, when predeparture testing was required, and June 12–September 3, 2022, when predeparture testing was not required. In a multivariable logistic regression model, pooled nasal swab specimens collected during March 20–June 11 were 52% less likely to be positive for SARS-CoV-2 than were those collected during June 12–September 3, after adjusting for COVID-19 incidence in the flight’s country of origin, sample pool size, and collection airport (adjusted odds ratio [aOR] = 0.48, 95% CI = 0.39–0.58) (p<0.001). These findings support predeparture testing as a tool for reducing travel-associated SARS-CoV-2 transmission and provide important real-world evidence that can guide decisions for future outbreaks and pandemics.

The Traveler-based Genomic Surveillance Program conducts surveillance of travelers at international airports for early detection of new and emerging SARS-CoV-2 variants and to fill gaps in international surveillance (*8*). International travelers aged ≥18 years arriving at airports in Newark, New Jersey (Newark Liberty Airport); New York, New York (John F. Kennedy International Airport); Atlanta, Georgia (Hartsfield-Jackson Atlanta International Airport); and San Francisco, California (San Francisco International Airport), who volunteered to participate provided a postarrival lower nasal swab sample in the airport (*8*). After providing signed consent, participants completed a standardized survey that included questions regarding demographic characteristics, flight country of origin, and whether predeparture testing had occurred and, if so, whether an antigen or molecular test had been performed. In the airport, dry nasal swab samples were pooled (5–25 samples per pool) by the flight country of origin. Pooled samples were sent to a laboratory in the Ginkgo Bioworks laboratory network for SARS-CoV-2 reverse transcription–polymerase chain reaction (RT-PCR) testing (*8*).

Postarrival RT-PCR testing results during March 20–June 11, when the predeparture test requirement was in effect, were compared with those during June 12–September 3, when predeparture testing was voluntary. To account for worldwide differences in COVID-19 incidence, pooled test results were matched with daily 7-day average country-level COVID-19 incidence (cases per 100,000 population) from the World Health Organization* based on pool collection date and the flight country of origin. To account for reporting differences by country, normalized incidence was estimated by dividing the 7-day average COVID-19 incidence on the date of pool collection for the flight country of origin by the maximum 7-day average daily incidence for that country during March 20–September 3, then multiplying by 100.

To identify factors associated with positive postarrival SARS-CoV-2 pooled test results, bivariate comparisons and univariable logistic regression were performed. Factors with significant univariable associations (p<0.05) were incorporated into a multivariable mixed effects logistic regression model that included collection airport as a random effect. Alternative periods (during the 4–8 weeks preceding June 12 and those on or after that date) were considered in sensitivity analyses. Analyses were conducted in R (version 4.0.2; R Foundation). This activity was reviewed by CDC and was conducted consistent with applicable federal law and CDC policy.^†^

During March 20–September 3, 2022, a total of 28,056 arriving travelers from 24 countries received testing for SARS-CoV-2, yielding 3,049 pooled samples with a median of eight participant samples per pool (range = 5–25). During March 20–June 11, among 16,668 Traveler-based Genomic Surveillance participants, 13,190 (79.1%) reported having had a predeparture test; during June 12–September 3, this percentage declined by 80% to 1,786 of 11,123 (16.1%) participants reporting having had a predeparture test (Figure 1). Among 14,976 participants who reported the type of predeparture test, 10,349 (69.1%) reported receiving an antigen test.

**FIGURE 1:**
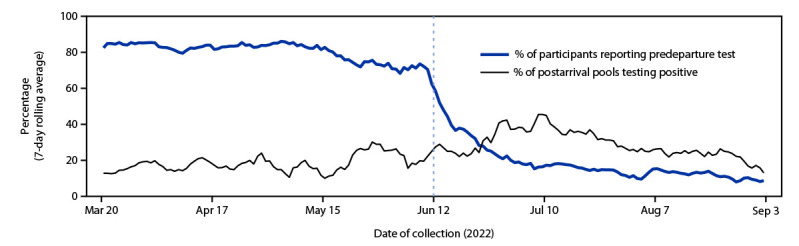
Percentages (7-day rolling average) of participants reporting a predeparture SARS-CoV-2 test* and pools^†^ testing positive for SARS-CoV-2^§^ during postarrival testing — Traveler-based Genomic Surveillance Program, United States, March 20–September 3, 2022 * Molecular or antigen test; a predeparture SARS-CoV-2 test was required for most travelers entering the United States before June 12, 2022. ^†^ In the airport, dry nasal swab samples from participants were pooled (5–25 samples per pool) by the flight country of origin. ^§ ^By reverse transcription–polymerase chain reaction.

During the analysis period, 691 (22.7%) of 3,049 sample pools tested positive for SARS-CoV-2 by RT-PCR. The percentage of positive pools increased 56% from 17.9% (291 of 1,622) during March 20–June 11, to 28.0% (400 of 1,427) during June 12–September 3 (p<0.001) (Figure 1) (Supplementary Table 1, https://stacks.cdc.gov/view/cdc/124584). The increase in the percentage of positive postarrival test results between March 20–June 11 and June 12–September 3 occurred across countries, collection airports, incidences, and pool sizes and was apparent in both bivariate analyses and univariable logistic regression. Participants during each period were similar in age and gender; however, during the period beginning June 12, fewer participants reported U.S. residency (Supplementary Table 2, https://stacks.cdc.gov/view/cdc/124585).

Multivariable model results showed that pools of samples collected during March 20–June 11 (when predeparture testing was mandatory) were 52% less likely to be positive than were those when predeparture testing was voluntary (aOR = 0.48, 95% CI = 0.39–0.58) (p<0.001), after adjusting for COVID-19 incidence in the flight’s country of origin, pool size, and collection airport (Table). COVID-19 incidence in the flight’s country of origin and pool size also remained significant predictors of positive pooled test results in the multivariable model.

**TABLE:** Unadjusted and adjusted mixed effects logistic regression results for postarrival pooled SARS-CoV-2 test results during 12-week windows before and after June 12, 2022 — Traveler-based Genomic Surveillance Program, four U.S. airports,* March 20–September 3, 2022

Variable (referent group)	Unadjusted		Adjusted	
	OR (95% CI)	p-value	OR (95% CI)	p-value
**Time window (Jun 12–Sep 3)**				
Mar 20–Jun 11	0.56 (0.47–0.67)	<0.001	0.48 (0.39–0.58)	<0.001
**Normalized incidence^†^ (0–20)**				
20–40	1.4 (1.1–1.8)	0.004	1.3 (1.0–1.6)	0.052
40–60	2.0 (1.5–2.6)	<0.001	1.8 (1.3–2.3)	<0.001
60–80	2.2 (1.6–3.1)	<0.001	2.1 (1.5–3.0)	<0.001
80–100	2.3 (1.8–3.0)	<0.001	2.2 (1.7–2.8)	<0.001
**Pool size (5–9 participants)**				
10–14	1.4 (1.1–1.6)	0.002	1.5 (1.2–1.9)	<0.001
≥15	1.4 (1.1–1.8)	0.004	2.6 (1.9–3.4)	<0.001

**Abbreviation:** OR = odds ratio.

* John F. Kennedy International Airport, New York, New York; Newark Liberty International Airport, Newark, New Jersey; Hartsfield-Jackson Atlanta International Airport, Atlanta, Georgia; and San Francisco International Airport, San Francisco, California.

^†^ Incidence was normalized by dividing the 7-day average COVID-19 incidence (cases per 100,000 population) for the flight origin country on the date of collection by the maximum 7-day average daily incidence for that country during the analysis period and multiplying by 100.

Sensitivity analyses were conducted by repeating the regression models using 4- and 8-week periods around June 12 and adjusting for the same covariates. Lower odds of positive test results before June 12 remained significant (4-week periods: aOR = 0.65, 95% CI = 0.44–0.96 [p<0.001]; 8-week periods: aOR = 0.48, 95% CI = 0.37–0.63 [p<0.001]) (Figure 2) (Supplementary Table 3, https://stacks.cdc.gov/view/cdc/124586).

**FIGURE 2:**
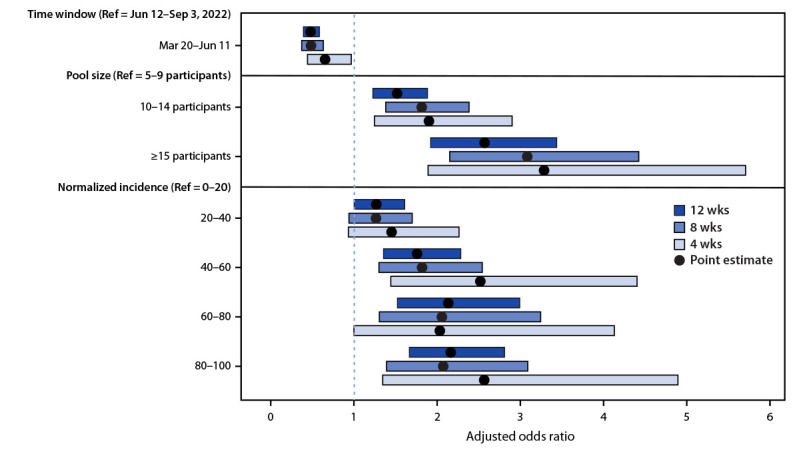
Comparison of mixed effects models*^,^^†^ for pooled SARS-CoV-2 test results across different time windows before and after June 12, 2022 — Traveler-based Genomic Surveillance Program, United States, March 20–September 3, 2022 **Abbreviation:** Ref = referent group. * Adjusted odds ratio point estimates relative to each Ref with 95% CIs. ^†^ Incidence was normalized by dividing the 7-day average COVID-19 incidence (cases per 100,000 population) for the flight origin country on the date of collection by the maximum 7-day average daily incidence for that country during the analysis period and multiplying by 100.

## Discussion

Postarrival SARS-CoV-2 test results were 52% less likely to be positive when the predeparture COVID-19 testing requirement was in effect than during the 12-week period after it was discontinued; this finding was true even when controlling for other factors such as incidence in the flight’s country of origin and pool size. These findings, based on observed, real-world traveler data, support the value of predeparture testing as a tool for reducing SARS-CoV-2 transmission associated with travel and were consistent with estimates from previous modeling studies (*4*–*7*).

Sensitivity analyses using shorter time frames around removal of the predeparture testing requirement produced similar findings. Although still statistically significant, the magnitude of this effect decreased when 4-week windows were considered, possibly because of smaller sample sizes during the shorter time frame or higher rates of voluntary predeparture testing during the 4 weeks after the removal of the predeparture test requirement on June 12.

The findings in this report are subject to at least five limitations. First, because participation in the Traveler-based Genomic Surveillance Program is voluntary, results might not be representative of all international travelers; however, any participation bias was likely consistent across both periods. Second, because of the pooled sampling and testing strategy employed, trends among individual participants could not be assessed. Third, incidence data were matched with pooled test results based on a flight’s country of origin, and it is possible that participants began their itinerary in a different country and later connected to the U.S.-bound flight. Fourth, as testing rates decline globally, reported incidence data might not fully reflect actual COVID-19 risk in a given country (*9*). Finally, not all travelers during March 20–June 11 had a predeparture test, such as those who recently had COVID-19 (*1*), and some travelers during June 12–September 3 voluntarily chose to test, potentially diminishing this estimate of the effect of predeparture testing.

Reducing the number of persons traveling while infected with SARS-CoV-2 through predeparture testing could reduce air travel–associated transmission in airports, aircraft, and destination communities. CDC continues to recommend testing before and after international travel (*3*). Along with other strategies, including isolation of persons with confirmed or suspected COVID-19 and masking, testing before international travel is an important element of a multipronged COVID-19 prevention strategy. In December 2022, results from this analysis were used alongside other evidence to support a predeparture test requirement for travelers boarding flights to the United States from China to slow importation of SARS-CoV-2 during a surge in COVID-19 cases there (*10*). These findings provide important real-world evidence supporting the effectiveness of predeparture testing that can guide decisions for future outbreaks and pandemics.

**Summary**

**What is already known about this topic?**

During December 6, 2021–June 11, 2022, SARS-CoV-2 testing ≤1 day before departure or proof of recent COVID-19 recovery were required for passengers boarding U.S.-bound flights. Mathematical models have estimated predeparture testing effectiveness in preventing travel-associated transmission.

**What is added by this report?**

CDC’s Traveler-based Genomic Surveillance Program collects postarrival nasal swabs for SARS-CoV-2 testing from volunteering international air travelers. Among 3,049 pooled (28,056 individual) samples collected during March 20–September 3, 2022, the predeparture testing requirement was associated with 52% lower postarrival SARS-CoV-2 positivity.

**What are the implications for public health practice?**

Predeparture testing can reduce SARS-CoV-2 transmission risk during or after travel by reducing the number of infectious travelers. These results can help guide decisions for future outbreaks.

## Footnotes

*
https://covid19.who.int/data

† 45 C.F.R. part 46.102(l)(2), 21 C.F.R. part 56; 42 U.S.C. Sect. 241(d); 5 U.S.C. Sect. 552a; 44 U.S.C. Sect. 3501 et seq.
